# Methodological Considerations Regarding the Comparison of Clevidipine and Nicardipine in Hypertensive Emergencies

**DOI:** 10.1002/clc.70476

**Published:** 2026-09-25

**Authors:** Shuai Zhang, Yishuo Zhang

**Affiliations:** ^1^ Baicheng Medical College Baicheng Jilin China; ^2^ Changchun University of Chinese Medicine Changchun Jilin China

Dear Editor,

1

We read with great interest the systematic review and meta‐analysis by Ullah et al. comparing clevidipine and nicardipine in hypertensive emergencies [1]. The authors have assembled the available comparative data on a question that clinicians face almost daily in emergency and critical care settings, and their conclusion that the two agents perform comparably on most efficacy and safety endpoints is broadly consistent with an earlier meta‐analysis restricted to cerebrovascular populations [2]. Several methodological aspects, however, deserve closer attention before these findings inform treatment choices.

First, eight of the nine included studies were retrospective, and pooling them with the single randomized trial in one random‐effects model may overstate the certainty of the pooled estimates. In observational cohorts, the choice between clevidipine and nicardipine is seldom arbitrary; it reflects illness severity, clinical context, institutional protocols, and formulary constraints, precisely the conditions under which confounding by indication can distort both efficacy and safety comparisons [3]. Newcastle–Ottawa scores were not uniformly high, and adjusted estimates from the cohorts were not synthesized separately. A design‐stratified analysis, or at minimum a sensitivity analysis restricted to the randomized data, would help readers judge how much of the comparable result reflects true equivalence rather than residual confounding.

Second, one of the primary outcomes, time to target systolic blood pressure, is not a standardized construct. The included studies used different target thresholds, titration intervals, and measurement frequencies, and the pooled estimate carried substantial heterogeneity (*I* ^2^ = 82%). The subgroup findings are also difficult to reconcile: clevidipine appeared faster in both clinical settings, yet the overall estimate did not reach statistical significance, and the difference in the general hypertensive crisis subgroup (−2.79 min) is unlikely to matter clinically. Subgroup‐specific estimates, without a formal test of interaction between subgroups, provide limited support for a genuine speed advantage. By contrast, the percentage of time within the target range, a measure of sustained control that has been linked to cardiovascular outcomes [4], did not differ between groups, which tempers enthusiasm for any meaningful pharmacologic superiority.

Third, the lower infusion volume associated with clevidipine should be interpreted cautiously. This difference largely reflects formulation: clevidipine is delivered as a concentrated lipid emulsion, whereas nicardipine is typically diluted in larger volumes. It is a pharmacokinetic and practical property rather than a demonstrated clinical benefit, and the high heterogeneity (*I* ^2^ = 88%) further limits interpretability. The authors' suggestion that lower infusion volume may reduce fluid overload is biologically plausible, and fluid accumulation is indeed associated with adverse outcomes in critically ill patients [5], but fluid balance was not among the outcomes captured by this analysis. The lipid load of clevidipine is a consideration in its own right, one that a meta‐analysis of this size is unlikely to capture.

In summary, we thank the authors for a useful synthesis. Analyses that stratify by study design, standardize outcome definitions, report formal interaction tests, and examine fluid balance directly would substantially strengthen the evidence base for choosing between these agents in hypertensive emergencies.

## Conflicts of Interest

The authors declare no conflicts of interest.

## Data Availability

Data sharing not applicable to this article as no data sets were generated or analyzed during the current study.
